# Cross-talk between androgen receptor and nerve growth factor receptor in prostate cancer cells: implications for a new therapeutic approach

**DOI:** 10.1038/s41420-017-0024-3

**Published:** 2018-01-31

**Authors:** Marzia Di Donato, Gustavo Cernera, Ferdinando Auricchio, Antimo Migliaccio, Gabriella Castoria

**Affiliations:** Department of Biochemistry, Biophysics and General Pathology, University of Campania ‘Luigi Vanvitelli’, Naples, Italy

Prostate cancer (PC) is the second leading cause of cancer death in men in Western society. Current therapies for PC remain, however, unsatisfactory and PC frequently progresses toward a castrate-resistant phenotype. Preclinical and clinical studies are aimed at investigating the molecular basis for PC progression^1^.

Nerve growth factor (NGF) belongs to the mammalian neurotrophin family of growth factors that control survival, differentiation, and neurite outgrowth. Neurotrophins also act on many different cell types, and deregulation of their signaling pathways is found in a number of tumors, including PC. Two classes of cell surface receptors, a family of tyrosine receptor kinases called Trks (TrkA, TrkB, TrkC) and the p75NTR receptor, mediate the effects of neurotrophins^2^. PC frequently synthesizes large amounts of NGF, which in turns stimulates TrkA^3^. Thus, TrkA might represent a promising target in PC therapies.

In this report, we show that androgens and NGF both induce a reciprocal cross-talk between androgen receptor (AR) and TrkA in prostate cancer-derived LNCaP cells. Such cross-talk is reminiscent of that observed in neuronal PC12 cells^4^, with significant differences in the final biological outcome, resulting on one hand in proliferation and migration of PC cells, and differentiation of PC12 cells on the other.

The non-aromatizable androgen R1881 robustly stimulates BrdU incorporation in LNCaP cells at levels only very slightly weaker than those triggered by serum. The AR antagonist bicalutamide inhibits this response, suggesting a role for AR in the observed response. Intriguingly, in addition to impairing the NGF proliferative effect, the TrkA inhibitor GW441756^5^ cross-inhibits the mitogenic effect of androgen. In turn, bicatulamide inhibits NGF action. Noteworthy, the stimulatory response by NGF is lower than that triggered by androgen (Supplementary Fig. 1A).

Cross-talk between AR and TrkA also regulates LNCaP migratory phenotype, as the cells respond to R1881 or NGF with a significant increase in cell motility, analyzed by Transwell (Supplementary Fig. 1B) or wound scratch (Supplementary Fig. 1C) assays, with a stronger stimulation by R1881 compared with that observed using NGF. Notably, the cross-inhibitory action of R1881 or NGF by GW441756 or bicatulamide, respectively, is also detected in these assays. Since androgen- or NGF-induced cell migration requires AR/filamin A association in various cell types, we also used a stapled peptide (RH2025u) specific for the site of interaction derived from the AR domain, which interrupts AR/filamin A complex assembly^4,6,7^. At nano-molar concentration, the peptide inhibits LNCaP cell migration triggered by androgens or NGF (Supplementary Fig. 1B and C), indicating a role for AR/filamin A complex in NGF-induced motility.

At last, we investigated whether the functional cross-talk observed in proliferation and migration responses is associated with co-immunoprecipitation of the two receptors. Supplementary Fig. 1D shows that treatment of LNCaP cells with R1881 or NGF triggers a specific co-immunoprecipitation of both receptors. Remarkably, association of AR with TrkA induced by R1881 is stronger than that induced by NGF. This difference correlates with the more robust functional effects triggered by androgens in proliferative and motility assays.

In sum, AR/TrkA reciprocal cross-talk enables the gain of proliferative and invasive properties of androgen- or NGF-challenged PC cells. Cross-inhibition of this complex by receptor antagonists impairs proliferative and migratory phenotype of these cells. Our results suggest that the combinatorial use of AR and TrkA inhibitors, commonly employed as single drugs, could be profitably tested in therapeutic trials for treatment of PC.

## Electronic supplementary material

Supplemental Figure 1

Supplementary information
