# Supplementary figures and images for: Cross-talk between androgen receptor and nerve growth factor receptor in prostate cancer cells: implications for a new therapeutic approach
Source: Cell Death Discov. 2018 Jan 31;4:5. doi: 10.1038/s41420-017-0024-3 (PMC5841355; doi:10.1038/s41420-017-0024-3)

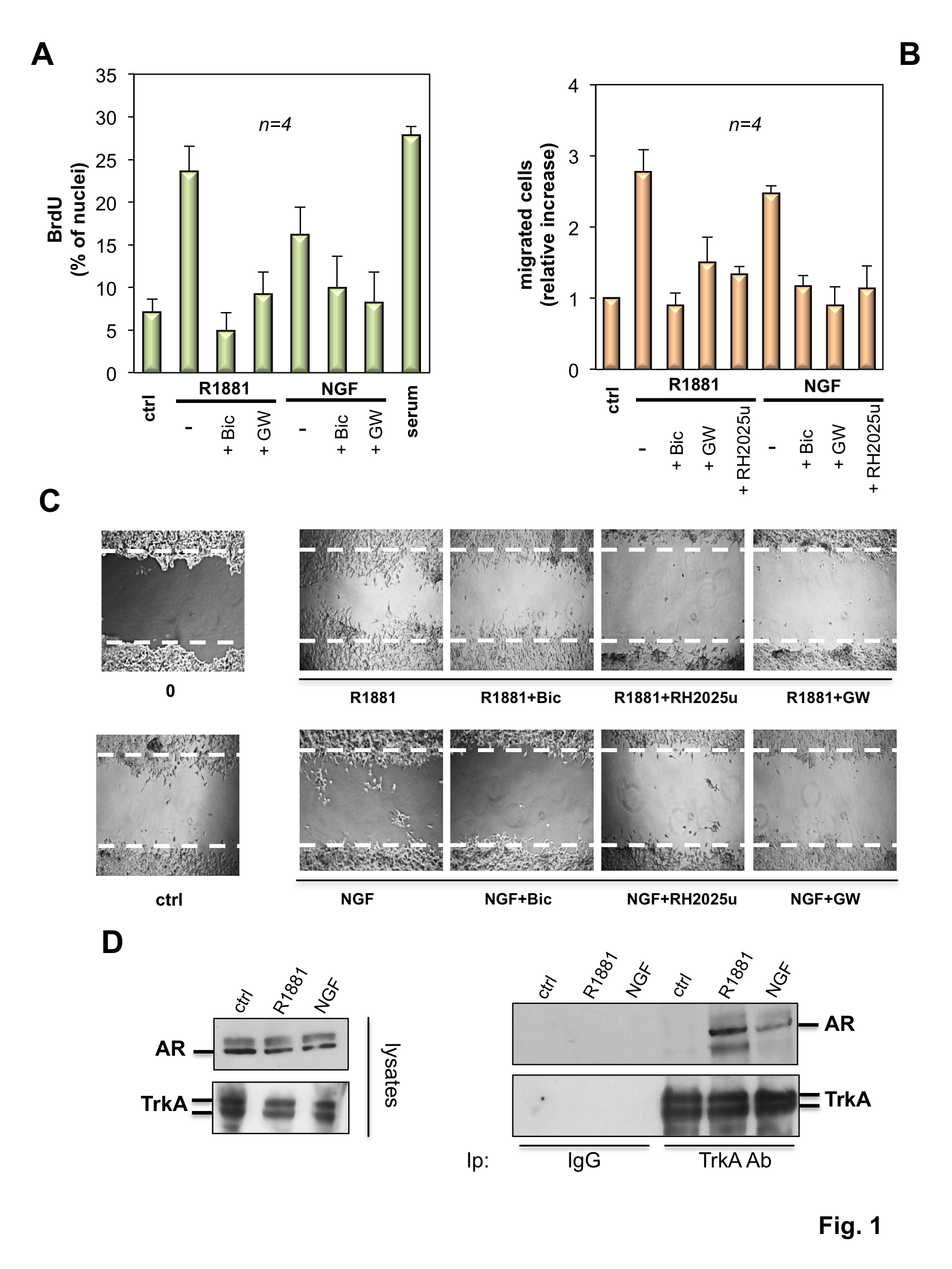

Supplement: Supplementary file 1 — Supplemental Figure 1 [file 41420_2017_24_MOESM1_ESM.tif]
