# Supplementary material for: Cross-talk between androgen receptor and nerve growth factor receptor in prostate cancer cells: implications for a new therapeutic approach
Source: Cell Death Discov. 2018 Jan 31;4:5. doi: 10.1038/s41420-017-0024-3 (PMC5841355; doi:10.1038/s41420-017-0024-3)
Supplement: Supplementary file 2 — Supplementary information [file 41420_2017_24_MOESM2_ESM.docx]

**Legend to Supplementary Figure 1.**

LNCaP cells were cultured and made quiescent as reported (7). They were then unchallenged or challenged with the indicated compounds. R1881 (Sigma) was used at 10 nM, NGF (Millipore) at 100 ng/ml, bicalutamide (Sigma) at 10 μM, [GW441756](https://www.ncbi.nlm.nih.gov/nuccore/GW441756) (Selleckem) at 1 μM, the RH2025u stapled peptide at 10 nM, and serum at 20%.

Quiescent LNCaP cells were used. **In A**, cells were left unstimulated (ctrl) or stimulated with the indicated compounds and then pulsed with 100 μM BrdU (Sigma) for 18 h. BrdU incorporation was analysed by immunofluorescence and expressed as a percentage of total nuclei, as previously described (7).  In **B**, cells were unstimulated (ctrl) or stimulated with the indicated compounds and then allowed to migrate on collagen-coated Transwells for 7 h. Migrated cells were stained with Hoechst and counted (6). Data are expressed as relative increase in number of migrated cells. In A and B, means and SEM are shown; *n* represents the number of experiments. In **C**, cells were wounded, then left unstimulated or stimulated with the indicated compounds and allowed to migrate for 48 h. Contrast-phase images were generated as described (6), and are representative of 3 different experiments, each performed in duplicate. In **D**, cells were unstimulated (ctrl) or stimulated with the indicated compounds for 10 min. In left panels, lysates were immunoblotted with anti-TrkA antibody (06-574; Millipore) or anti-AR antibody (N-20; Santa Cruz Biotechnology). As previously described (8), the anti-TrkA antibody recognizes two different isoforms. The Western blot for AR also reveals a further band, in addition to the 110K Da immune-reactive band. This band migrates slowly and might represent a product of post-translational modifications of AR (4). *In right panels*, lysate proteins were immunoprecipitated with anti-TrkA or control (IgG; Pierce) antibodies. Proteins in immune complexes were analysed using anti-AR antibody.
